# Supplementary material for: CheXmask: a large-scale dataset of anatomical segmentation masks for multi-center chest x-ray images
Source: Sci Data. 2024 May 17;11:511. doi: 10.1038/s41597-024-03358-1 (PMC11101488; doi:10.1038/s41597-024-03358-1)
Supplement: Supplementary file 1 — Supplementary Information [file 41597_2024_3358_MOESM1_ESM.pdf]

Supplementary: Statistical analysis for Max RCA-estimated DSC scores

In the main manuscript we included statistical analysis for Mean RCS-estimated DSC scores since this indicator showed better correlation with real DSC. However, for the sake of completeness, here we include the same analysis for the alternative Max RCA-estimated DSC score.

Table 1. Dice RCA (Max) statistical analysis

Dataset name	Sample size (n)	Mean	Std	Min	1%	5%	25%	50%	75%	Max
Chestx-ray8	112120	0.882	0.044	0.164	0.732	0.806	0.864	0.890	0.909	0.967
CheXpert	187825	0.870	0.038	0.415	0.757	0.801	0.849	0.875	0.896	0.961
MIMIC-CXR-JPG	243334	0.873	0.050	0.179	0.690	0.784	0.854	0.884	0.904	0.968
Padchest	96184	0.893	0.042	0.128	0.714	0.831	0.882	0.901	0.917	0.970
VinDr-CXR	18000	0.892	0.035	0.501	0.778	0.831	0.876	0.898	0.915	0.964

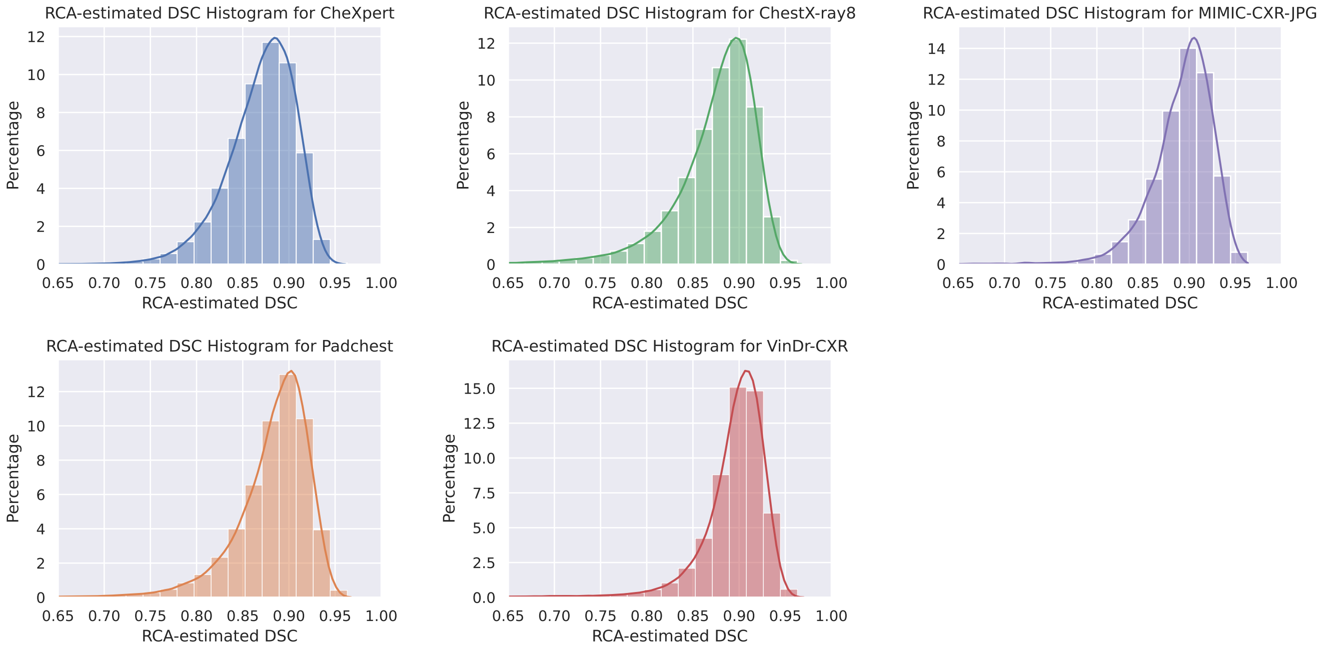

Figure 1. Histograms illustrating the distribution of segmentation quality across the five datasets. Each histogram represents the distribution of RCA (Reverse Classification Accuracy) estimations of DSC for a specific dataset using the Max value instead of the mean, providing a visual representation of the segmentation performance. The histograms are truncated at 0.65 for visualization purposes. The full range of values is considered in the statistical analysis (Table 1).
